# Unsupervised learning-based approach for detecting 3D edges in depth maps

**DOI:** 10.1038/s41598-023-50899-3

**Published:** 2024-01-08

**Authors:** Ayush Aggarwal, Rustam Stolkin, Naresh Marturi

**Affiliations:** Extreme Robotics Lab, School of Metallurgy and Materials, University of Birmingham, Edgbaston, UK

**Keywords:** Computer science, Information technology

## Abstract

3D edge features, which represent the boundaries between different objects or surfaces in a 3D scene, are crucial for many computer vision tasks, including object recognition, tracking, and segmentation. They also have numerous real-world applications in the field of robotics, such as vision-guided grasping and manipulation of objects. To extract these features in the noisy real-world depth data, reliable 3D edge detectors are indispensable. However, currently available 3D edge detection methods are either highly parameterized or require ground truth labelling, which makes them challenging to use for practical applications. To this extent, we present a new 3D edge detection approach using unsupervised classification. Our method learns features from depth maps at three different scales using an encoder–decoder network, from which edge-specific features are extracted. These edge features are then clustered using learning to classify each point as an edge or not. The proposed method has two key benefits. First, it eliminates the need for manual fine-tuning of data-specific hyper-parameters and automatically selects threshold values for edge classification. Second, the method does not require any labelled training data, unlike many state-of-the-art methods that require supervised training with extensive hand-labelled datasets. The proposed method is evaluated on five benchmark datasets with single and multi-object scenes, and compared with four state-of-the-art edge detection methods from the literature. Results demonstrate that the proposed method achieves competitive performance, despite not using any labelled data or relying on hand-tuning of key parameters.

## Introduction

Visual features such as keypoints, edges, color, and texture are essential for many computer vision and robotics applications, such as scene registration and visual servoing. Edge features are of particular interest due to their versatility in various fields, especially where extracting other features is challenging. For instance, teaching a robot to disambiguate objects in industrial environments can be challenging, due to many metallic objects and surfaces, which are monochrome, smooth and textureless. With the proliferation of 3D vision sensors and their increasing use in robotics and other fields like medicine and construction, we believe an efficient technique to estimate edges in 3D data is indispensable. To this end, we propose a learning-based 3D edge detection method that extracts edges from depth maps constructed from organized point clouds.

3D edges are characterised by the points with recognisable variation or depth discontinuities. Specifically, these are the points (on the surface) that represent the separation between volumetric regions. These 3D edge features contain interesting geometric information about the objects in the scene and assist in differentiating the useful foreground details from the background. They can be used as the basis for many tasks like object detection and tracking^1^, object full pose estimation and registration^2^, scene characterisation and segmentation for grasping^3^, human action recognition^4^, surface reconstruction^5^ etc. In general, there are three type of 3D edges: occluded edges, occluding edges and high curvature edges. Occluded edges represent the boundary points on the background surface, which are generated due to occlusion by a foreground object. Occluding edges represent the surface boundaries on the objects present in the scene. Lastly, high curvature edges are the edge points along abrupt variations on the object surfaces. In this paper, we focus on estimating occluding and high curvature edges in depth maps, as these are of significant interest in many applications like robotic grasping, manipulation, segmentation, etc.

2D edge detection has been extensively investigated since the early years of computer vision research, and a plethora of robust 2D edge detectors have been developed and utilised for a variety of tasks. In contrast, the research on 3D edge estimation remains relatively limited. Although the process of edge estimation in 3D is similar to that of 2D, the two-dimensional detectors cannot sufficiently hand the complex 3D data. Most of the 2D edge detectors work by calculating gradients in the local area of each image point^6^. The greater the variation, the higher the gradient and hence, such points are classified as edge points. Practically, this is performed by convolving the image iteratively with a mask. These gradient-based approaches provide a good mathematical solution to detect edges. 3D vision data encodes the information about scene depth and is relatively larger in size than 2D images. Unlike in 2D images where edges are given by changes in pixel intensity or colour values, the 3D edge estimation process corresponds to finding the depth discontinuities and changes in the surface orientations. Calculating the gradients and finding the local maxima for all three gradient directions for all the points is computationally intensive^7^. Furthermore, selecting an appropriate gradient threshold for designating edges is a difficult problem, and this threshold value decision greatly impacts the quality of the detected edges. Some previous studies have selected these thresholds via statistical analysis of the data^8^, but this requires extensive manual tuning. We note that each depth map is unique, and produces gradients in different ranges. For this reason, a single threshold is not optimal for a variety of data samples. In order to resolve this issue, we adopt a deep learning-based feature extraction method, which captures generic features from the input depth maps. These features are then used for detecting edges. Over the past decade, deep learning-based approaches have become popular for solving similar image processing problems. Most of the available approaches use supervised learning, wherein extensive ground-truth labelled datasets are required a-priori to learn an optimal solution^9^. However, having such labelled datasets is not always possible, especially in practical applications.

In this paper, we present a deep learning-based unsupervised method for 3D edge detection and parameter selection, utilising both learning-based and gradient edge features. We identify edges by clustering each point in the depth map as either edge or non-edge. Majority of the available learning-based approaches are majorly equipped with learning components and occasionally use non-learning feature extraction (e.g., extracting edges, corners, lines etc.) as a pre-processing step^9^. Nevertheless, to the best of our knowledge, exploring non-learning based feature extraction as an integral part of the network learning has not been reported in the literature. Specifically, to extract 3D edges, we have designed our network as a multi-stage architecture comprising of the following components: (i) encoder–decoder; (ii) multi-size split; (iii) edge feature extractor; and (iv) cluster network. The encoder–decoder module allows the network to learn and extract intrinsic features within the processed input data. These features are then split into multiple sizes to obtain insights about the input at different abstraction levels. The edge feature extractor then extracts the edge features for all these input sizes. This extractor is a non-learning component, which is designed to capture gradients from the learned features and to further filter out the noise. The extracted edge features are then fused and passed through a clustering block where the edge points are distinguished from the non-edge points via unsupervised learning. Additionally, we propose a non-learning pre-processing algorithm to filter and complete the missing depth information (shadows) in the input depth data. The major advantage of the proposed method is that it is independent of manual threshold fine-tuning and labelled data requirements, while achieving competitive performance as state-of-the-art methods. In the proposed network architecture, deep learning-based layers achieve generality across a wide variety of different data, while feature extraction provides guided feature learning specific to the task. This type of combined network can be used to detect different features by changing the feature extraction layers, and can be readily adapted for different applications. We demonstrate the edge detection capability of our proposed method by comparing its performance with four state-of-the-art methods using five publicly available benchmark datasets. The detailed analysis of results, along with the description of datasets and compared methods is discussed in the experiments section.

## Related work

Edge detection is a thoroughly explored concept in literature^10^, initially devised for identifying edges within 2D images. However, with the recent commercialisation and increased affordability of 3D sensors, novel algorithms tailored for 3D edge detection have emerged. Presently, state-of-the-art methods can be categorised into two types: feature-based and learning-based algorithms, which are discussed below.

### Feature-based 3D edge detection methods

Early attempts at 3D edge detection involved evaluating surface curvatures and performing neighbour searches on object model meshes and point clouds^11,12^. Despite providing refined edges of 3D models, they are often time-consuming. Bormann et al. proposed a fast method^13^ for detecting edges in organised point clouds by finding the depth and surface discontinuities. They pre-processed input depth data and filtered depth image over various parameters that are adjusted based on dataset and sensor errors. A contour detection algorithm^14^ is presented by Hackel et al. for unorganised point clouds using surface properties at the local neighbourhood of each point. Choi et al.^2^ proposed a 3D edge detection method on organised point clouds exploiting indexed neighbour property. Their method finds four different types of edges, including RGB edges, occluding edges, occluded edges, and high curvature edges with distance and angle thresholds tuned for the datasets. A modified canny-based method to detect edges in depth maps is proposed by Sung et al.^15^ wherein, at first an edge preserving smoothing is performed and then morphological open and close operations are performed after canny detection. Apart from these methods, some other feature-based edge detection algorithms are also proposed^7,16–18^. While effective, these methods can be sensitive to noise and produce spurious edges. Furthermore, they often require meticulous parameter tuning, posing challenges in practical implementation. Consequently, learning-based methods have gained popularity in recent years for edge detection.

### Learning-based 3D edge detection methods

These types of methods extract or learn distinguishable features from the data to differentiate edge points from non-edge ones. Bode et al. introduced an edge detection network for 3D point clouds^19^, focusing on leveraging local neighborhood statistics for substantial geometric structures such as buildings. Hu et al. proposed a joint semantic segmentation and semantic edge detection deep network^20^, integrating features from semantic segmentation and detected edge outputs to refine edge detection. Kaneko et al. introduced a binary decision-tree-based edge detection method^21^, employing gradient depth images (derived from Sobel filters) and their corresponding ground truths for model parameter learning. Sarkar et al. developed a deep learning-based occluding edge detection method^22^ that evaluates small image patches to classify center pixels as edge or non-edge, utilizing a threshold-based edge detection method for creating training ground truth data, establishing a lower bound on attainable error. Guerrero et al. proposed a supervised depth contour prediction method^23^, employing a multi-channel input (RGB, depth, and normal) and an encoder–decoder-based convolutional neural network (CNN) for edge learning. In general, these learning-based methods rely on having labeled ground truth data, which isn’t consistently accessible. Additionally, the task of generating a precisely labeled dataset is both time-consuming and costly.

### Unsupervised learning

The concept of unsupervised learning has been proposed in the literature to understand intrinsic patterns within the data^24^. It has been widely used for image processing and analysis to perform tasks like classification^25^, anomaly detection^26^, etc. Previously, some studies have investigated the use of unsupervised learning for 2D edge estimation^27,28^. A recursive neural network (RNN)-based method is presented by Le et al. to detect edges in natural images^27^. An encoder–decoder is integrated with a feedback loop to improve the predicted edge images. Li et al. utilised video input alongside computed flow images for 2D edge detection, generating intermediate expected edges using the flow image gradients^28^. To the best of our knowledge, no methods using unsupervised learning for 3D edge estimation are reported in the literature. In this work, we incorporate unsupervised learning by employing clustering on embedded deep parameters to categorise data into edge or non-edge classes.

### Clustering

It is an unsupervised grouping technique that has been extensively studied for diverse problem domains^29–31^. Various methods drive clustering, including aligning data points based on factors like feature means of similar samples (e.g., K-means), similar probability distribution, distance-based or density-based matching^30^. Recently, deep learning-based clustering has emerged^29,32^, where neural networks are utilised to learn a clustering-friendly representation. Techniques such as deep autoencoder-based (DAE) clustering, deep neural network-based (DNN) clustering, and graph neural network-based (GNN) clustering have been explored^29^. Within these, we specifically focus on the DAE-based clustering methods. Xie et al.^33^ employed k-means clustering on a deep embedding, minimising changes in cluster means across epochs. Wang and Jiang^34^ proposed an unsupervised deep clustering method using adaptive Gaussian Mixture Model (GMM) modeling and optimisation, enhancing intra-cluster compactness and inter-cluster separability. Guo et al.^35^ proposed a self-paced learning-based deep clustering approach with augmentations to the data. The network is trained in two stages, i.e., first the features are learned through an encoder–decoder network and then the network is fine-tuned through clustering. Affeldt et al.^36^ and Yang et al.^37^ proposed methods integrating spectral clustering with deep neural networks to achieve domain aligning embedding. In the proposed work, we adopt clustering from Xie et al.^33^ and devise a joint solution for model training.

## Methods

Developed 3D edge detection methodology is presented in this section. In a given point data, edges can be detected using features like gradients in *X* and *Y* directions, surface normal at each point in the point cloud, geometric properties of the neighbourhood, etc. As mentioned before, processing these features and further grading the respective points as edge or non-edge often requires manual fine-tuning of various thresholds. This is not only tedious but may inherently introduce errors. Also, fixing a threshold might not yield optimal results over the complete range of dataset. Hence, we propose a clustering-based automated threshold selection approach to achieve generality over given data. Before presenting the technical details of our method, in the following section, we first introduce the problem in detail.

### Problem formulation

Given a depth map $\mathscr {D}$ of size $W \times H$ (corresponding to an organised point cloud) with $\textbf{D}$ points, we aim to find a threshold $\lambda$ to detect the edge points $\textbf{E}_p \subset \textbf{D}$, where each edge point ${e}_{i,j} \in \textbf{E}_p$ at location $i \in \{1, 2,\ldots , W\}$ and $j \in \{1, 2,\ldots , H\}$ in the depth map is given as:

1 $$\begin{aligned} {e}_{i, j} = {\left\{ \begin{array}{ll} {d}_{i,j}, &{}\quad \text {if } \Delta _{i,j} > \lambda \\ 0, &{}\quad \text {otherwise} \end{array}\right. }, \end{aligned}$$

where, ${d}_{i,j}$ and $\Delta _{i,j}$ is the depth value and edge feature at $\{i, j\}$-th index, respectively. The optimal threshold $\lambda$ belongs to the edge feature distribution $\varvec{\Gamma }$ obtained from features calculated at all the points $\textbf{D}$ in all the depth maps of the dataset. We have considered an unsupervised learning-based *k-means* clustering approach to learn two clusters with means $m_1, m_2$ in $\varvec{\Gamma }$ as non-edge and edge regions, respectively. Using these means, $\lambda$ is defined as:

2 $$\begin{aligned} \lambda = \mathop {\mathrm {arg\,min}}\limits _{\varvec{\Gamma }} \{ |(m_1-\varvec{\Gamma })|> |(m_2-\varvec{\Gamma })|\} \end{aligned}$$

For more details on using unsupervised clustering for deep learning applications, we refer the reader to work by Fuller^32^. For computing the edges, in this work, we have adapted the gradient-based features $\Delta _{i,j}$ as defined in Eq. (3).

3 $$\begin{aligned} \Delta _{i,j}= & {} \sqrt{(\textbf{G}_X * \textbf{D}_{i,j})^{2} + (\textbf{G}_Y * \textbf{D}_{i,j})^{2}} \end{aligned}$$

where, $\textbf{G}_{X}$ and $\textbf{G}_{Y}$ are the gradient operators to evaluate the depth change by applying convolution operation ($*$), on local region matrix $\textbf{D}_{i,j}$, which is given as

4 $$\begin{aligned} \textbf{D}_{i,j} = \begin{bmatrix} d_{i-1, j-1} &{}\quad d_{i-1,j} &{}\quad d_{i-1, j+1} \\ d_{i, 
j-1} &{}\quad d_{i,j} &{} d_{i, j+1} \\ d_{i+1, j-1} &{}\quad d_{i+1,j} &{}\quad d_{i+1, j+1} \end{bmatrix} \end{aligned}$$

**Figure 1:**
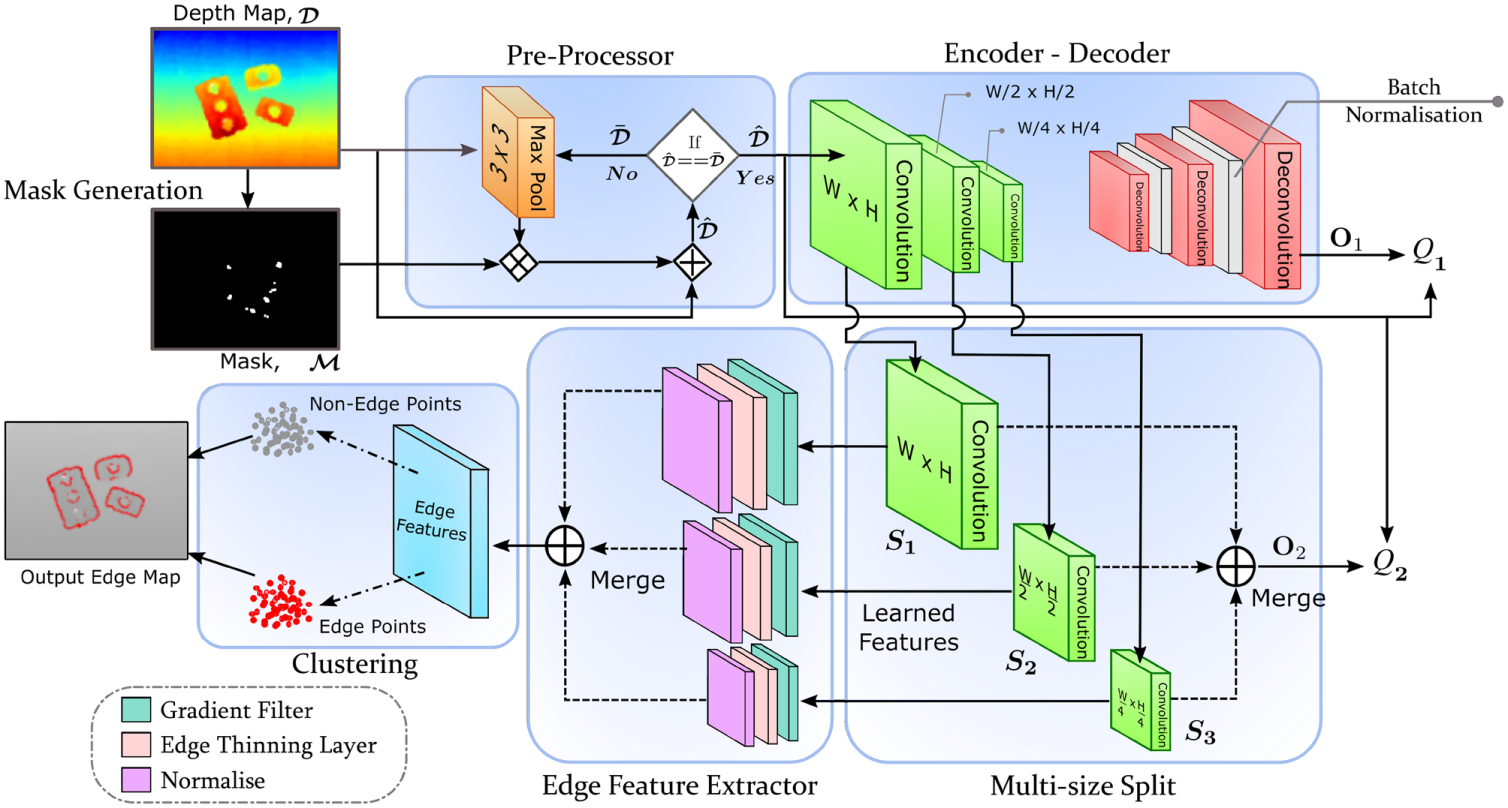
Proposed DNN model for unsupervised 3D edge detection.

It is worth noting that these features can be replaced with any other edge detection features. To facilitate unsupervised clustering, we have incorporated the edge feature extraction with deep neural network (DNN) based learning. The proposed network architecture to accomplish 3D edge detection in depth images is depicted in Fig. 1. Overall network is categorised into two major components, learning-based and feature-based. Learning-based components include encoder–decoder network and clustering sub-network. These learn features from the data. Feature-based components include pre-processing and edge feature extractor. These are responsible for calculating pre-defined features from the data. Detailed description of each of these components, same order as in the network shown in Fig. 1, is presented below.

### Pre-processing

Most of the depth sensors suffer from empty information patches in the captured depth data. This happens due to shadows, or reflective or absorbing surfaces^38,39^. The presence of such shadows can affect the performance of edge detection algorithms as points near these empty patches will show very high discontinuities, causing confusion in learning a threshold. Hence, we propose a pre-processing algorithm to eliminate empty patches from the input depth maps. It’s important to note that the final output of the network is consistent with the original input depth map and does not include edges in the empty regions.

Previously, various shadow removal algorithms are proposed in the literature. Zhang and Funkhouser proposed a deep learning-based depth map completion method^39^; however, they have used colour images in support of depth map to perform the task. A low gradient regularisation algorithm to in-paint missing depth information is proposed by Xue et al.^40^. A depth map in-painting technique using light field epipolar plane image geometry is proposed by Yang et al.^41^. Both these techniques provide good performance but are slow for online or real-time applications. A morphology-based algorithm for shadow removal from depth map was presented by Danciu et al.^38^. We propose a similar but simpler shadow removal method that uses max depth feature selection. It uses a maxpool layer with a pool size of $3 \times 3$ to determine the maximum depth value in local regions of the depth map. The pool size is considered as the radius of the local region. Larger pool sizes reduce overall pre-processing time, but may amplify random noise in the data. Conversely, smaller pool sizes are not as affected by noise but may increase processing time. Our analysis across a wide range of depth maps shows that the $3$ filter size provides a reasonable trade-off.

**Figure 2:**
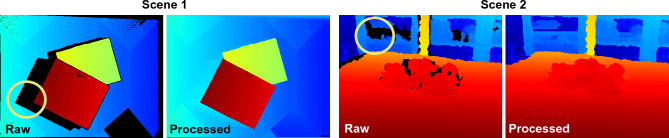
Illustration of pre-processing process for two different scenes. Sample shadow regions are marked by circles.

Our method’s flow graph is shown in the in the pre-processor stage of Fig. 1. It takes the depth map and its corresponding mask as inputs. The mask is generated by identifying all the empty regions in the depth map. Depth map is first passed through the maxpool layer, and then its output is element-wise multiplied () with the mask. Later, the resulting map is element-wise added () to the input depth map. This process is repeated until no further change can happen. The resulting pre-processed depth map is considered as the input to the next stage. Sample results illustrating the performance of our pre-processor are shown in Fig. 2. with this method, the average pre-processing time per image is approximately 20–30 ms.

### Encoder–decoder DNN architecture

An encoder–decoder deep network is used to learn intrinsic features from the data^27,28,33^. In order to learn such relationships from the input depth maps, we consider a convolution and deconvolution based three-stage encoder–decoder network with three side outputs $S_1, S_2,$ and $S_3$, as shown in Fig. 1. This side output like structure is inspired by the work of Xie and Tu^42^, who presented a holistically-nested edge detection (HED) method to extract 2D edges.

The encoder is designed as a three layer convolution with each subsequent layer half the size of previous layer. For all the encoding layers, a kernel size $3 \times 3$ is considered with channel sizes (16, 16, 16) in subsequent layers, respectively. Size of the input is reduced in the subsequent layers by using a stride of 2. The stride is used in the convolution layer instead of the maxpool layer due to the inverse nature of depth values in a depth map, i.e., background has higher depth than foreground object. After convolution, a ReLu activation function is used to achieve non-linearity gain. The three encoding layer outputs are taken as the side outputs, over which further convolution operation is performed to create a single channel feature map. These side outputs provide learned features at three different scales of the original depth map, i.e., $\{1\times , 0.5\times , 0.25\times \}$. The side outputs help in detecting edges at different level of abstraction. As each side output is half the size of the previous one, the collected features at smaller sizes provide an abstracted view of the scene. This abstraction improves the detected edges around the object boundaries. In contrast to that of in HED, we do not train for individual side outputs; instead, we fuse them using deconvolution upscaling layers and let the network learn essential features for each scale inherently.

With decoder, we have used three deconvolution layers to upscale the encoded features from the encoder and then output a map of same size as the input. Each deconvolution layer is considered with a kernel size of $3 \times 3$ and (16, 16, 1) channels, respectively. ReLu activation function is used to get the output activation except for the last output layer, where a sigmoid activation function is used. To prevent over-fitting and to accelerate the training, batch normalisation layers are considered after first and second deconvolution layers as shown in Fig. 1. Since no annotated data is required for learning, we train our encoder–decoder network in a self-supervised manner. The learning is performed by minimising the mean square errors (MSE) ${Q}_{1}$ and ${Q}_{2}$, which are respectively computed at the outputs $\textbf{O}_1$ and $\textbf{O}_2$ using the pre-processed depth map $\widehat{\mathscr {D}}$ as target. These two errors, also termed as losses, are calculated as:

5 $$\begin{aligned} \begin{aligned} {{Q}_{1}} = \frac{1}{WH}\sum _{i}^{W}\sum _{j}^{H}{\left[ \textbf{O}_1(i,j) - \widehat{\mathscr {D}}(i,j)\right] }^{2} \qquad {{Q}_{2}} = \frac{1}{WH}\sum _{i}^{W}\sum _{j}^{H}{\left[ \textbf{O}_2(i,j) - \widehat{\mathscr {D}}(i,j)\right] }^{2} \end{aligned} \end{aligned}$$

The ${Q}_{1}$ loss is used to train the layers of the encoder–decoder block and achieve generalised learning over the training data. On the other hand, the ${Q}_{2}$ loss is employed to prevent the side outputs from losing information about the input scene structure during the learning process. Both of these losses calculate the mean square difference between the pre-processed input and the corresponding outputs of the network. The error gradients, which are calculated using these losses, are back-propagated to train the encoder–decoder and multi-size split blocks.

### Edge feature extractor

As mentioned previously, the edge feature extractor is a non-learning feature extraction layer proposed in our network. Specifically, this layer captures the edge features from the multi-size side outputs of the encoder–decoder. It is a dynamic component in our network as its main purpose is to capture application specific features (in our case they are edge features), along with the learned features in the model.

The proposed edge feature extractor consists of three stages, as seen in Fig. 1. The first stage contains a gradient filter, which is implemented as a convolution operation with a predefined kernel for both height *H* and width *W* directions in the depth map. Four different kernels are studied in this work: Sobel, Roberts, Prewitt, and Laplacian of Gaussian (LoG). Their performance is discussed later in the experiments section. The output of this stage, i.e., the magnitude of the gradient is then passed through the next stage, an edge thinning layer. Here, a minimum-pool filter with a selected kernel size is applied to remove noisy edges detected by the gradient filter and also to thin down the gradients around the edges. Following this, the data is normalised using *min–max* normalisation. As seen in Fig. 1, the edge features are learned on each of the side outputs of the encoder–decoder network to detect edges at different scales. After this, we upscale all the extracted edge features to the input map size and fuse them. Finally, a single edge-feature map is produced as an output of this layer.

### Learning-based clustering

A deep embedded clustering approach is considered to perform unsupervised classification of the features learned from the network. Within this, a stochastic gradient descent based learning is performed using back-propagation on the cluster objective function to optimise the cluster parameters. In this work, we have used *k-means* clustering to perform clustering of the edge features extracted at all the points in the depth image. The problem considered in this paper can be categorised as a binary classification problem aiming at finding for each point if it is an edge or not. Following this, we define the number of clusters as two and evaluate a soft probability for each point in the edge feature map to belong to one of the clusters using student-T distribution, as in Eq. (6).

6 $$\begin{aligned} p_{i,j}^{k} = \frac{(1 + {|| \Delta _{i,j} - m_{k} ||}^{2}/\alpha )^{-\frac{\alpha +1}{2}}}{\sum _{k'}(1 + {|| \Delta _{i,j} - m_{k'} ||}^{2}/\alpha )^{-\frac{\alpha +1}{2}}} \end{aligned}$$

where, $\{i,j\}$ represents the coordinates of a point on the edge feature map, $p_{i,j}^{k}$ is the soft-probability of a point to belong to the *k*-th cluster. $k \in \{1,2\}$ in our problem where, when $k=1$, then $k'=2$ and vice versa. $m_{k}$ and $m_{k'}$ are the cluster means being learned by the layer. $\alpha$ is the degree of freedom component, to decide the impact of Euclidean distance between the mean and a given point. For experiments, $\alpha =1$ is used. To facilitate unsupervised learning of the algorithm, a temporary target probability distribution *T* is computed at the start of each epoch over the complete training set. This target distribution is determined by aggregating the network’s predictions across the entire training dataset to generate a prediction matrix $\textbf{U}$. Using this matrix, per class frequency $\textbf{f}$ is calculated by summing the predictions for each class. As in (7), $\textbf{f}$ is subsequently used to compute the per sample, per point target probability *T* for each class, which serves as the basis for training.

7 $$\begin{aligned} {\textbf{f} = \sum _{i=1}^{B}\sum _{j=1}^{W}\sum _{k=1}^{H}{} \textbf{U}_{i, j, k}} \quad \quad {T = \frac{\textbf{U}/\textbf{f}}{\sum _{i=1}^{2}{\textbf{U}/\textbf{f}}}_{i}} \end{aligned}$$

With each iteration in the epoch, a difference between current distribution *C* and target probability *T* is computed using the KL-Divergence loss (KL-Divergence loss calculates the difference between two distributions by calculating the average distance required to overlap the current distribution with target distribution^43^), as:

8 $$\begin{aligned} {\textrm{KL}} = {T} *\log ({T}/{C}) \end{aligned}$$

Minimising KL-Divergence loss maximises the difference between the Gaussian distributions in a given probability density function, hence increasing the space between the non-edge and edge point distributions. Gradients are calculated using Eq. (8), which are back-propagated through the layers for learning. Hence, The total loss *Q* used to perform the network learning is given by,

9 $$\begin{aligned} {{Q} = {\textrm{KL}} + {{Q}_{1}} + {{Q}_{2}}} \end{aligned}$$

The output of the clustering layer is an edge map with each point classified as either edge or non-edge based on the soft probabilities. Assuming that the objects are majorly present in the centre of the sensor view, in this work, we consider a centred masking layer before the clustering layer. The prime benefit of using this layer is that it removes the unwanted background noise from the features before clustering to learn better edges. It is worth noting that this masking is optional and is only used for experimentation. It is not an integral part of our network.

### Network model discussion

In this section, we present a discussion on the evolution of the proposed network design from concept to realisation. The proposed network model is obtained after multiple experiments over the layer configurations. Initially, we have considered a feature extractor and clustering combined network architecture to detect edges on single size of input depth map. Though this simple network performed the task passably, it was unable to generalise over variations in the 3D data. Moreover, it failed to capture the edges around the structure of the object. To tackle these issues, encoder–decoder layers with three side outputs are added before the feature extraction layer. These encoder–decoder layers helped achieve generic learning over the dataset and the three side outputs helped to improve the detection of edges around the object structure. Although the learning of the three side outputs can be performed individually, to prevent extra processing, we have decided to train them by combining them together into original image size. Merging of side outputs is experimented with upscaling and deconvolution operations. Using upscaling, we notice that the smaller scale side outputs are unable to learn features properly. Hence, we chose deconvolution, which was effective in learning the features in the side outputs.

In edge feature extractor, the kernel size of the edge thinning layer is selected empirically after testing multiple kernels of sizes $\{1,2,5,7\}$. The kernel with size 1 indicates no edge thinning operation is performed. Although it provided all the gradients, it was susceptible to noise and network performance dropped. For higher kernel sizes of 5 and 7, it is observed that some of the required gradients vanished in the process and accordingly, a portion of the edges are not detected. Eventually, we have selected a kernel with size 2 for our final model. Merging of edge features extracted at the side outputs is another decision point where both upscaling and deconvolution can be used. For merging edge features, upscaling is selected to preserve the gradient values in the merged image and then further perform clustering on the same. Concisely, following this aforementioned analysis, we have decided upon the final network presented in Fig. 1. Detailed evaluation results of our network are discussed in the next section.

## Results

We conducted experiments on five benchmark datasets to demonstrate the effectiveness of our approach across noiseless and noisy real-world sensor data. We compared the performance of our method with four state-of-the-art methods: Choi et al.^2^, fast edge^13^, Sung et al.^15^, and JSENet^20^, by evaluating the poses obtained by registering the edge points onto the object model. We divided the performance analysis into two parts. First, we performed pose registration and F-measure comparison on single-object scenes. Next, we evaluated the performance on multi-object scenes following similar analysis.

### Dataset description

The five datasets used for evaluation are Tejani et al.^44^, T-LESS^45^, PartNet^46^, NYU^47^, and MvTech-ITODD^48^. In Tejani et al. dataset, the training sets contain depth maps of various household objects. These are synthetically generated; hence, noise-free. The T-LESS dataset contains depth sensor collected real-world data of industrial objects (both single and multi-object). PartNet dataset contains point clouds of various indoor objects, which are part-level annotated and labelled by means of semantic segmentation. From this, we have generated ground truth labelled depth maps for our work. NYU dataset contains depth maps of 464 indoor room scenes, with multiple occluded objects. MvTech-ITODD dataset comprises of real-world multi-object depth maps of industrial objects captured with a depth sensor. From Tejani et al., T-LESS, and PartNet datasets, we have considered 5000 depth maps distributed over different objects and scenes for training and 500 depth maps for testing following the same distribution. 1200 depth maps from T-LESS multi-object scenes and NYU dataset have been considered for training and 250 depth maps for testing. From MvTech-ITODD dataset, we have considered 800 depth maps for training and 150 depth maps for testing. Training and testing sets are mutually exclusive. For analysis, we consider Joystick, Juice Carton, Milk, Camera, Cup, and Shampoo objects from Tejani et al.; from T-LESS, we have used objects with ID-numbers 2, 5, 8, 17, and 27; and from PartNet, Knife, Scissors, Bowl, Bottle, and Mug are used. From NYU, depth maps from all 464 scenes are used, and from MvTech-ITODD, depth maps of all 28 objects have been used.

### Experimental setup

Our network is implemented using Tensorflow library in python. We have trained our network on a single Nvidia Tesla P100 GPU. While training, network requires 32 GB RAM and 16 GB GPU memory. High RAM requirements are due to intensive target distribution calculation and can grow with increasing data. For a depth map of size $640 \times 480$ points, training time is approximately 4 hours per dataset. Excluding the input loading time, the network takes about $60 - 80$ ms (including pre-processing) to compute the output edge map. The inference model requires very less space and can be run on systems with $< 2$ GB GPU memory and 4 GB RAM.

The thresholds used with Choi et al.^2^, Fast Edge^13^ and Sung et al.^15^ methods are represented by $\lambda _{c}$, $\lambda _{f}$ and $\lambda _{s}$, respectively. Each threshold contains two values. For $\lambda _c$, the first value is the depth discontinuity and the second one is the maximum neighbour search. For $\lambda _f$, the first value represents the depth step factor and the second value is the minimum edge angle threshold. For $\lambda _s$, first is sigma in color and the second is sigma in coordinate space for the smoothing filter. Thresholds for these compared methods are selected from the range of values provided in their respective papers. Some tests are conducted on a subset of the considered datasets with different combinations of these threshold values. The final thresholds are selected based on their average performance over all the objects in that respective dataset. JSENet^20^ is trained over PartNet^46^ and NYU^47^ datasets for 200 epochs with no further variation in network training parameters.

### Evaluation metrics

Pose registration and F-measure analysis are used to compare the performance of our method with state-of-the-art methods. Poses are computed by performing point cloud registration between the detected edge points and the respective object 3D model, using random sample consensus (RANSAC). The rotations and translations of the resulting homogeneous transformation matrix are used for evaluation. The following performance metrics are computed for quantitative analysis: (i) $||{A}||_F$—Frobenious norm of the difference in registered and ground truth transformation matrices; (ii) $\textbf{R}_{err}$—rotation error; (iii) $\textbf{T}_{err}$—translation error in terms of Euclidean distance; and (iv) $\varvec{Z}_E$—average distance (ADD) of two model points transformed by registered transformation and ground truth transformation, respectively. F-measure helps analysing accuracy of our method against labelled ground truth edge data. This metric is used only for PartNet^46^ and NYU^47^ datasets since they are the only ones with labeled data. To calculate F-measure, soft precision and recall are derived based on the distance thresholds between predicted and ground truth edge points. The distance threshold used to calculate soft precision and recall is one neighbour point distance in all the directions. Among these metrics, for $||{A}||_F$, $\textbf{R}_{err}$, $\textbf{T}_{err}$, and $\varvec{Z}_E$, smaller values indicate better performance, whereas larger values are preferable for the F-measure, soft precision, and recall. These metrics are calculated for all the samples within the testset. Note that the results shown for qualitative analysis are chosen based on the optimal performance of the proposed method, irrespective of the performance of the compared methods.

### Comparison analysis: single object scenes

**Figure 3:**
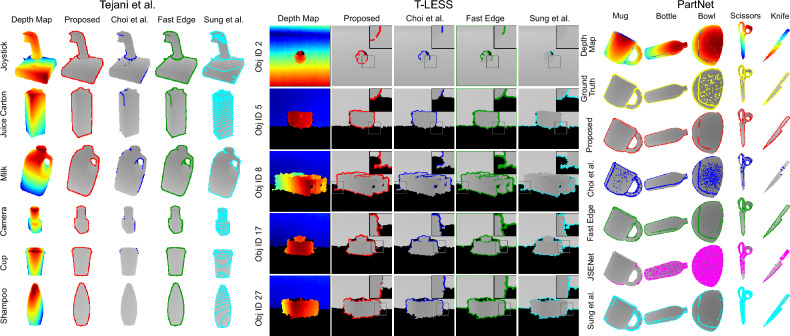
3D edge detection analysis for single object scenes with Tejani et al.^44^, T-LESS^45^, and PartNet^46^ datasets. Note that for T-LESS dataset, a zoom-in region is shown on the top right corner of each image to highlight important comparison details.

Pose registration analysis for Tejani et al.^44^, T-LESS^45^, and Partnet^46^ datasets are summarised respectively in Tables 1 and 2. The detected edges of some sample objects from all the datasets are shown in Fig. 3. As JSENet is a supervised learning method, it is evaluated only with PartNet dataset. Table 3 shows the performance comparison of our method with state-of-the-art methods in terms of precision, recall and F-measure averaged over the entire PartNet dataset.

#### With Tejani et al. dataset^44^

Our proposed method performed comparably to Choi et al.^2^, Fast Edge^13^, and Sung et al.^15^. Overall, our method achieved the best translation performance for most of the objects with relatively less error. On average, all the three state-of-the-art methods show higher standard deviation, indicating that the selected hyper-parameters are sub-optimal for all depth maps in the dataset and require further fine-tuning to achieve better performance. Furthermore, it is observed that Choi et al.^2^ method is not able to detect all edges effectively and Sung et al.^15^ also detected surface points as edges for multiple objects. On the other hand, our method is able to correctly detect object edges. Overall, these results clearly highlight our method’s ability and efficiency in estimating 3D edges. For this test, $\lambda _c = (0.05, 50)$, $\lambda _f = (0.05, 40)$, and $\lambda _s = (75, 75)$ are used.

#### With T-LESS dataset^45^

Our method performed comparably to all the state-of-the-art methods across all objects, while achieving the best result for ADD errors. From the results, it can be inferred that our method exhibits satisfactory performance on noisy real-world data, without requiring any parameter tuning as needed by the compared methods. It is evident from Fig. 3, where our method shows substantially better edge detection. On the other hand, Choi et al.^2^ is not able to identify the edges on the lower side of the objects while Fast Edge^13^ and Sung et al.^15^ not only detected all object edges but also marked the background noise as edges, e.g., edges are detected at the shadow boundary. Comparatively, our method performed well by not detecting nearby noisy regions as edges. For this test, we have used $\lambda _c = (0.02, 30)$, $\lambda _f = (0.02, 30)$, and $\lambda _s = (75, 75)$.

#### With PartNet dataset^46^

Our method outperformed all the compared methods in terms of mean error, while also exhibiting good performance in terms of standard deviation for translation and ADD errors. Also, it is comparable to other methods for rotation and pose difference metrics. From Fig. 3, Choi et al.^2^ is not able to identify all edges, whereas JSENet^20^ and Sung et al.^15^ detected non-edge points as edges. However, our proposed and Fast Edge^13^ methods displayed good performance and detected edges comparable to the ground truth. This is evident from the Table 3. Our method recorded a high precision and F-measure among all, and exhibited close performance for recall with Fast Edge^13^ method. Higher precision indicates that the detected edges correctly match with the ground truth labels. Experiments are conducted using $\lambda _c = (0.02, 40)$, $\lambda _f = (0.02, 40)$ , and $\lambda _s = (75, 75)$.

**Table 1:** Pose registration analysis with Tejani et al.^44^ and T-LESS^45^ datasets.

M	Tejani et al.^44^					T-Less^45^				
	Object	$||{A}||_F$*	$\textbf{R}_{err}$*	$\textbf{T}_{err}$*	$\varvec{Z}_E$*	Obj.ID	$||{A}||_F$*	$\textbf{R}_{err}$*	$\textbf{T}_{err}$*	$\varvec{Z}_E$*
Choi et al.^2^	Joystick	**2.42** ± 0.64	2.13 ± 0.74	0.66 ± 0.26	0.11 ± 0.05	2	**2.53** ± 0.53	**2.19** ± 0.65	0.79 ± 0.33	0.02 ± 0.01
	Juice	2.54 ± 0.54	2.31 ± 0.68	0.65 ± 0.24	0.09 ± 0.04	5	**2.45** ± 0.77	2.19 ± 0.87	0.80 ± 0.37	0.04 ± 0.02
	Milk	2.27 ± 0.73	2.01 ± 0.84	0.58 ± 0.26	0.10 ± 0.06	8	**2.43** ± 0.91	**2.28** ± 0.99	0.77 ± 0.40	0.08 ± 0.05
	Camera	2.33 ± 0.73	**2.09** ± 0.85	0.60 ± 0.28	0.05 ± 0.03	17	**2.32** ± 0.81	2.03 ± 0.89	0.74 ± 0.40	0.05 ± 0.03
	Cup	2.30 ± 0.63	**1.98** ± 0.72	0.59 ± 0.24	0.06 ± 0.03	27	**2.47** ± 0.80	2.27 ± 0.91	**0.80** ± 0.39	0.08 ± 0.04
	Avg.	2.37 ± 0.65	2.10 ± 0.76	0.62 ± 0.26	0.08 ± 0.04	Avg.	**2.44** ± 0.76	2.19 ± 0.86	**0.78** ± 0.38	0.05 ± 0.03
Fast Edge^13^	Joystick	2.42 ± 0.58	**2.08** ± 0.69	0.70 ± 0.27	0.10 ± 0.05	2	2.57 ± **0.47**	2.18 ± **0.61**	0.90 ± 0.30	0.02 ± 0.01
	Juice	2.48 ± 0.64	2.25 ± 0.77	0.67 ± 0.26	0.09 ± **0.02**	5	2.55 ± **0.55**	**2.19** ± **0.66**	0.87 ± 0.33	0.04 ± **0.01**
	Milk	**2.24** ± 0.78	**1.99** ± 0.88	0.56 ± 0.26	**0.09** ± 0.06	8	2.60 ± **0.79**	2.42 ± **0.86**	0.89 ± 0.40	0.08 ± 0.05
	Camera	2.58 ± 0.56	2.39 ± 0.70	0.68 ± 0.26	0.06 ± 0.03	17	2.46 ± **0.66**	2.12 ± 0.76	0.84 ± **0.36**	0.05 ± 0.02
	Cup	2.43 ± 0.56	2.13 ± 0.69	0.64 ± 0.22	0.06 ± 0.03	27	2.50 ± 0.76	2.26 ± 0.86	0.85 ± 0.40	0.08 ± 0.04
	Avg.	2.43 ± 0.62	2.17 ± 0.74	0.65 ± 0.25	0.08 ± 0.04	Avg.	2.53 ± **0.65**	2.23 ± **0.75**	0.87 ± 0.36	0.05 ± **0.02**
Sung et al.^15^	Joystick	2.46 ± 0.53	2.17 ± 0.68	0.62 ± 0.28	0.11 ± **0.03**	2	**2.53** ± 2.72	3.14 ± 3.0	**0.76** ± 2.74	0.03 ± 0.01
	Juice	**2.30** ± 0.56	**2.00** ± 0.70	**0.47** ± 0.23	0.08 ± **0.02**	5	2.64 ± 3.14	2.24 ± 0.71	**0.80** ± 3.19	0.04 ± 0.01
	Milk	2.49 ± 0.53	2.21 ± **0.67**	0.64 ± 0.27	1.11 ± **0.03**	8	2.52 ± 1.65	3.14 ± 3.0	**0.68** ± 1.62	0.08 ± 0.02
	Camera	**2.32** ± 0.59	2.05 ± 0.74	**0.5** ± 0.23	0.05 ± **0.01**	17	2.55 ± 1.78	2.26 ± **0.70**	**0.69** ± 1.76	0.05 ± 0.01
	Cup	**2.29** ± 0.58	2.00 ± 0.73	**0.45** ± 0.21	0.06 ± 0.02	27	2.65 ± 3.68	**2.16** ± **0.72**	0.86 ± 3.74	0.08 ± **0.02**
	Avg.	**2.33** ± 0.57	**2.03** ± 0.72	0.55 ± 0.21	**0.06** ± **0.02**	Avg.	2.65 ± 3.68	**2.16** ± **0.72**	0.86 ± 3.74	0.08 ± 0.02
Ours	Joystick	2.46 ± **0.47**	2.19 ± **0.64**	**0.53** ± **0.20**	**0.10** ± 0.05	2	2.56 ± 0.51	2.20 ± 0.68	0.90 ± **0.28**	**0.02** ± **0.01**
	Juice	2.44 ± **0.50**	2.18 ± **0.67**	**0.53** ± **0.20**	**0.08** ± 0.04	5	2.51 ± 0.63	2.20 ± 0.75	0.84 ± **0.33**	**0.04** ± 0.02
	Milk	2.49 ± **0.52**	2.31 ± 0.71	**0.50** ± **0.20**	0.11 ± 0.05	8	2.55 ± 0.86	2.40 ± 0.93	0.87 ± **0.38**	**0.08** ± **0.05**
	Camera	2.50 ± **0.48**	2.27 ± **0.66**	0.56 ± **0.17**	**0.05** ± 0.02	17	2.33 ± 0.80	**2.02** ± 0.88	0.80 ± 0.37	**0.04** ± **0.02**
	Cup	2.47 ± **0.48**	2.24 ± **0.65**	0.47 ± **0.17**	**0.06** ± **0.02**	27	2.49 ± **0.72**	2.24 ± 0.84	0.84 ± **0.36**	**0.08** ± 0.04
	Avg.	2.47 ± **0.49**	2.23 ± **0.66**	**0.52** ± **0.18**	0.07 ± 0.03	Avg.	2.48 ± 0.70	2.21 ± 0.81	0.85 ± **0.34**	**0.05** ± **0.02**

$^{*}$Smaller values indicate better performance. Significant values are in bold.

**Table 2:** Pose registration analysis with PartNet dataset^46^.

	Object	$||{A}||_F$*	$\textbf{R}_{err}$*	$\textbf{T}_{err}$ *	$\varvec{Z}_E$*		$||{A}||_F$*	$\textbf{R}_{err}$*	$\textbf{T}_{err}$*	$\varvec{Z}_E$*		$||{A}||_F$*	$\textbf{R}_{err}$*	$\textbf{T}_{err}$*	$\varvec{Z}_E$*
Choi et al.^2^	Knife	3.06 ± 1.14	2.15 ± 0.97	1.84 ± 1.27	2.49 ± 0.56e1	JSENet^20^	1.47 ± 1.36	1.54 ± 1.50	0.14 ± 0.24	0.75 ± 0.17e1	Ours	**1.45** ± 1.37	**1.54** ± 1.50	**0.10** ± **0.14**	**0.72** ± **0.15e1**
	Scissors	2.75 ± 1.19	2.04 ± 1.09	1.54 ± 1.13	7.01 ± **0.58e1**		1.74 ± 1.27	1.77 ± 1.41	0.24 ± 0.32	6.27 ± 0.95e1		**1.72** ± 1.22	**1.73** ± 1.38	**0.22** ± 0.24	**5.44** ± 0.62e1
	Bowl	3.05 ± 0.81	2.16 ± **0.70**	1.74 ± 1.06	9.78 ± **1.08e1**		1.89 ± 0.86	1.64 ± 0.92	0.25 ± 0.2	8.31 ± 1.42e1		**1.75** ± 1.00	**1.49** ± 0.95	**0.24** ± 0.55	8.12 ± 1.38e1
	Bottle	2.99 ± 1.07	2.09 ± 0.81	1.69 ± 1.29	8.61 ± **0.80e1**		2.03 ± 0.87	1.85 ± 0.99	0.24 ± 0.22	1.24 ± 2.68e1		**1.93** ± 0.96	**1.77** ± 1.06	**0.23** ± 0.26	**1.09** ± 3.76e1
	Mug	2.98 ± 0.77	2.15 ± **0.70**	1.66 ± 0.97	1.11 ± 1.63e1		1.85 ± 1.60	1.68 ± 1.16	0.29 ± 1.24	**1.01** ± **1.14e1**		**1.53** ± 1.20	**1.46** ± 1.26	**0.16** ± **0.14**	8.27 ± 1.20e1
	Avg.	2.96 ± 0.99	2.12 ± **0.70**	1.69 ± 1.14	7.79 ± **0.93e1**		1.79 ± 1.19	1.69 ± 1.19	0.23 ± 0.44	7.56 ± 1.27e1		**1.67** ± 1.15	**1.59** ± 1.00	**0.19** ± 0.26	**6.69** ± 1.42e1
Fast Edge^13^	Knife	2.69 ± 1.31	1.83 ± 1.13	1.65 ± 1.22	1.63 ± 0.23e1	Sung et al.^15^	2.23 ± **0.65**	2.02 ± **0.77**	0.40 ± 0.17	5.3 ± 2.7e1					
	Scissors	2.71 ± 1.10	1.96 ± 1.05	1.50 ± 1.08	7.04 ± 0.79e1		2.31 ± **0.63**	2.14 ± **0.77**	0.4 ± **0.16**	6.2 ± 2.4e1					
	Bowl	2.99 ± 0.84	2.15 ± **0.70**	1.65 ± 1.04	**1.10** ± 3.09e1		2.29 ± **0.56**	2.08 ± 0.71	0.38 ± **0.17**	9.4 ± 2.7e1					
	Bottle	2.62 ± 0.88	2.01 ± 0.86	1.24 ± 0.90	9.58 ± 1.98e1		2.26 ± **0.60**	2.03 ± **0.72**	0.39 ± **0.18**	7.2 ± 2.6e1					
	Mug	2.87 ± 0.80	2.09 ± 0.75	1.55 ± 0.93	1.35 ± 2.81e1		2.17 ± **0.68**	1.96 ± 0.79	0.38 ± 0.16	8.0 ± 2.6e1					
	Avg.	2.77 ± 0.98	2.00 ± 0.89	1.52 ± 0.70	8.55 ± 1.78e1		2.24 ± **0.63**	2.03 ± 0.76	0.39 ± **0.17**	7.4 ± 3.0e1					

*Smaller values indicate better performance. Significant values are in bold.

**Table 3:** Performance analysis of the proposed method with PartNet dataset^46^.

Method	Choi et al.^2^	JSENet^20^	Fast Edge^13^	Sung et al.^15^	Ours
Precision*	0.16	0.08	0.63	0.37	**0.82**
Recall*	0.19	0.20	**0.90**	0.84	0.84
F-measure*	0.14	0.10	0.72	0.48	**0.81**

*Larger values indicate better performance. Significant values are in bold.

### Comparison analysis: multi object scenes

**Figure 4:**
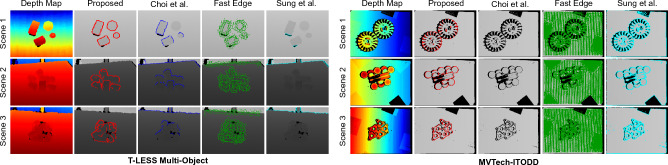
3D edge estimation analysis with T-LESS^45^ multi-object and MVTech-ITODD^48^ datasets.

**Figure 5:**
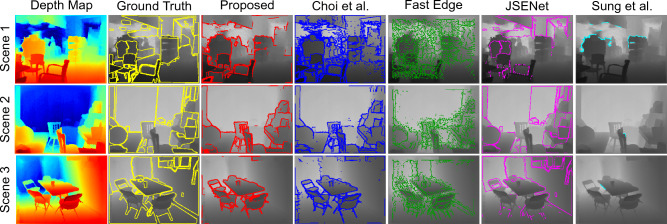
3D edge estimation analysis with NYU^47^ dataset.

Edge detection results for multi-object scenes from T-LESS^45^, MvTech-ITODD^48^ and NYU^47^ datasets are presented in Tables 4 and 5. Figure 4 shows detected 3D edges for different scenes from T-LESS multi-object^45^ and MvTech-ITODD^48^ datasets with all methods (except JSENet^20^), and Fig. 5 shows edges detected for scenes from NYU^47^ dataset for all the methods along with the ground truth edges provided with the respective datasets. Pose registration analysis is performed by estimating all individual object poses in each scene and by taking the mean value. Edges of each object in the scene are segmented out using the provided ground truth masks with the respective datasets. These results clearly validate the suitability of our method for (unstructured) multi-object scenarios. Experiments are conducted using $\lambda _c = (0.02, 40)$, $\lambda _f = (0.05, 40)$, and $\lambda _s = (75, 75)$.

**For T-LESS Multi-Object**^45^ **scenes,** we have observed that the proposed method outperforms all the compared methods for all the metrics. From Fig. 4, it can be seen that Choi et al.^2^ is able to detect partial edges on the objects while Fast Edge^13^ detected a lot of noise around the object as edges. Sung et al.^15^ method detect edges around the shadow regions instead of on the object. However, our proposed method is able to detect good 3D edges on the objects with no edges from the background.

**For MvTech-ITODD**^48^ **scenes,** the proposed method outperformed the compared methods for all metrics. In Fig. 4, with the used parameters, it is observed that Choi et al.^2^ did not detect any edges for some samples while Fast Edge^13^ method detected surrounding noise also as edges. Sung et al.^15^ is able to detect the object edges, but also detects shadow regions as edges. In comparison, our proposed method displayed good edge detection and registration performance without using any ground truth or hyper-parameter tuning.

**For NYU scenes,** due to the lack of ground truth poses for different objects in the scene, we do not perform pose analysis. However, we analyse F-measure performance for all the methods. From Table 5, it can be seen that the proposed method showcases best performance among all the methods, which means that our method achieve higher generalisation towards unseen samples and the detected edges are closer to the ground truth edges. In the samples shown in Fig. 5, Choi et al.^2^ and Fast Edge^13^ methods are able to detect the edges around the objects, but also detected a lot of noise on flat surfaces as edges. JSENet^20^, with supervised learning, is able to detect most edges, but the edges are broken and incomplete. Overall, the proposed method outperformed all the compared methods for multi-object scenes without requiring any labelled data and parameter tuning.

**Table 4:** Performance analysis for multi-object scenes with T-LESS^45^ and MvTech-ITODD^48^ datasets.

Metric	T-Less^45^				MvTech ITODD^48^			
	Ours	Choi et al.^2^	Fast Edge^13^	Sung et al.^15^	Ours	Choi et al.^2^	Fast Edge^13^	Sung et al.^15^
$||{A}||_F$*	**2.21**	2.31	2.41	2.23	**2.45**	2.53	2.80	2.52
$\textbf{R}_{err}$*	**1.85**	1.93	2.03	1.91	**2.10**	2.27	2.54	2.20
$\textbf{T}_{err}$*	**0.82**	0.90	0.94	0.85	**0.69**	0.71	0.72	0.79
${\varvec{Z}}_E$*	0.046	**0.044**	0.047	0.048	**0.044**	0.047	0.058	0.051

*Smaller values indicate better performance. Significant values are in bold.

**Table 5:** Performance analysis for multi-object scenes with NYU^47^ dataset using ground truth edges.

Method	Choi et al.^2^	JSENet^20^	Fast Edge^13^	Sung et al.^15^	Ours
Precision*	0.31	0.48	0.33	0.61	**0.59**
Recall*	0.39	0.24	0.34	0.01	**0.44**
F-measure*	0.33	0.38	0.31	0.02	**0.49**

*Larger values indicate better performance. Significant values are in bold.

## Contribution discussion: with practical analysis

In this section, we will analyze and discuss the effectiveness of various components in our proposed edge detection method. Furthermore, we have evaluated the computational complexity of our network and examined the real-world performance of our method. For further detailed results, please refer to the supplementary video.

### Generic network structure

**Figure 6:**
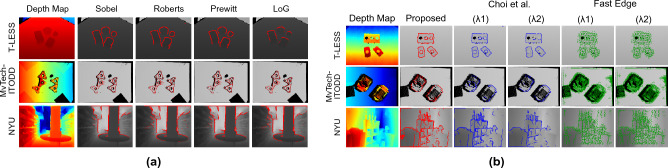
(**a**) Performance evaluation of the proposed network with different edge feature extractors. (**b**) Analysing the effect of thresholds on the performance of the compared methods with the proposed method.

In our proposed network architecture, the feature extraction layer is generic and can be modified to identify different features. We validate this behaviour by considering different edge feature extractors and observe the performance of our method. To this extent, we have considered four different gradient kernels: Sobel, Roberts, Prewitt, and Laplacian of Gaussian (LoG)^49^, and model them as individual edge feature extractors. The performance comparison of our method considering these edge feature extractors is shown in Fig. 6a. It is observed that the LoG filter is not able to detect all the edges and is also susceptible to noise. Overall, Sobel edge feature extractor is observed to perform well for all the considered datasets. Further, as a part of our design testing, we have trained a model considering all the four kernel features, concatenated together, for clustering. However, no impact over the performance of the network is observed. It is worth noting that all the kernels extract gradients from the depth map and collectively provide similar kind of features. Hence, concatenating them have no impact on the prediction.

### Benefit of automatic parameter selection

One of the key features of our method is the independence from threshold fine-tuning while achieving generality to the data. We evaluate the effect of thresholds on the performance of Choi et al.^2^ and Fast Edge^13^ by varying their threshold values. For Choi et al., $\lambda _{c,1} = (0.05, 50)$ and $\lambda _{c,2} = (0.02, 30)$ are used and for Fast Edge, $\lambda _{f,1} = (0.05, 40)$ and $\lambda _{f,2} = (0.02, 30)$ are used. These values are selected based on the results reported in their respective articles. Figure 6b shows the performance of Choi et al.^2^ and Fast Edge^13^ with these thresholds against our method. We see that small change in thresholds significantly affect the overall performance of Choi et al.^2^ method. While for Fast Edge^13^ method, there is no change due to threshold variation and is still detecting noise around objects as edges. Our method, after learning the threshold via unsupervised learning, showcase comparable performance to the best results of the state-of-the-art.

### Need for pre-processing

**Figure 7:**
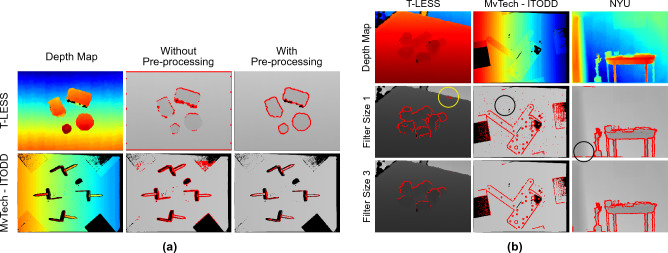
(**a**) 3D edge detection performance of the proposed method without and with pre-processing of input depth image. (**b**) Effect of the edge thinning kernel size on our network performance.

As stated earlier, existence of the empty shadow regions in the depth maps, especially when captured by real depth sensors, can inhibit the edge extraction process. Here, we discuss the necessity and effectiveness of the developed pre-processing approach. As shown in Fig. 7a, it is clearly seen that the edges detected without pre-processing are mainly centred around the empty shadows as these points have highest depth variations. However, most of the points on the object boundary, which actually represent edges, are clearly not detected. Nevertheless, when pre-processing is enabled, the method correctly detected the edges that actually belong to the objects and not on the shadow areas.

**Figure 8:**
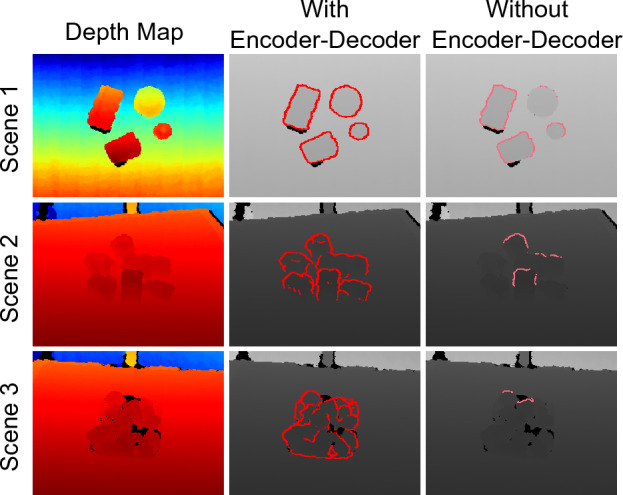
Performance evaluation of the proposed method without encoder–decoder module for T-LESS multi-object dataset.

### Effects of edge thinning

Edge thinning operation is part of the edge feature extractor layer in our proposed network. Within it, the edge features are passed through a minimum pool kernel. It is mainly used to thin down the gradients around edges as well as to remove unwanted noise. As shown in Fig. 7b, the one with smaller filter size detected all edges properly for all the datasets but it is prone to background noise, while larger kernel size produced incomplete or broken edges. Therefore, after multiple tests by experimentation, we have used a kernel with size 2 at each scale for all the previously reported experiments.

### Encoder–decoder requirement

As presented, the proposed method employs an encoder–decoder-based feature extraction before running the edge feature extraction module. The objective of this feature extractor is to learn intrinsic features from the input, aiming for consistent learning across input variations. To verify this, a separate network is trained without the encoder–decoder module to predict edges across the T-LESS Multi-object dataset. The corresponding results are depicted in Fig. 8. Analysis of the results reveals that the network trained without the encoder–decoder fails to detect complete edges. Moreover, its performance lacks consistency across different scenes, indicating an inconsistent cluster threshold learned by the network due to dataset variations. This observation underscores the importance of incorporating the encoder–decoder for enhanced generic learning in edge detection within our proposed method.

### Analysing the effect of central masking

**Figure 9:**
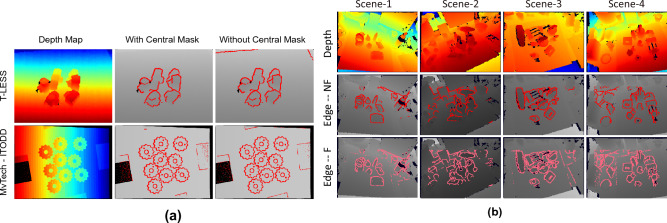
(**a**) Analysing the effect of central masking. (**b**) 3D edges detected using proposed method for four different scenes from Stefan et al.^50^ dataset. “Edge—NF” represents the results when no fine-tuning is performed, while “Edge—F” are with fine tuning of a trained model.

As mentioned earlier, we have used an optional central masking layer before clustering to filter the background noise caused due to abrupt depth variations. Here, we have conducted experiments with and without this masking layer to analyse its effect on our network performance. As shown in Fig. 9a, no significant effect has been observed by removing this layer for sample scenes from MvTech-ITODD dataset, due to pre-processing filling out all the noise in the depth map. While for T-LESS dataset, for very few instances, abrupt depth changes due to noise are detected as edges, which are masked out when central masking is enabled. Nevertheless, removing central masking has only a minimal effect on the overall edge detection performance.

### Generalisation to new data

To verify the adaptability of our trained model to entirely new scenes, we conducted experiments using the new Stefan et al.^50^ dataset. We conducted two tests for real-world multi-object scenes: one without fine-tuning (Edge—NF) and the other with fine-tuning (Edge—F). For each case, we used a set of 100 randomly selected depth maps from the dataset. For Edge—F, The model is fine-tuned for 20 epochs. Obtained results are shown in Fig. 9b for four different scenes. It is observed that both Edge—NF and Edge—F perform comparably, with Edge—F demonstrating slightly better performance due to extra fine-tuning. Consequently, these results highlight that the proposed model can detect edges for a completely new dataset without requiring additional training or parameter tuning, showcasing its ability for generalisation in learning.

### Complexity analysis

**Figure 10:**
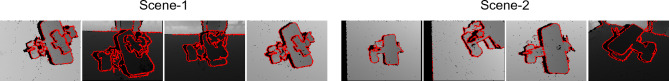
Screenshots from live 3D edge detection using proposed method.

In general, the conventional 3D edge detectors work by convolving a mask of size *M* to estimate the gradients in all directions. For 3D data of size, $\left( X\times Y \times Z\right)$, convoluting by a mask of size $\left( M\times M \times M\right)$ takes $\mathscr {O}\left( M^3 * size(X) * size(Y) * size(Z)\right)$ operations^7^. If the data is dense, this complexity is even higher. This not only restricts the size of the mask to be used for data of different sizes, but also compromises the accuracy of edge estimation. On the other hand, the computational complexity of our inference model is computed to be $\mathscr {O}(HW + H + W)$ where *W* and *H* are respectively the width and height of the input depth map. Full derivation is provided in the supplementary file. It is calculated by considering the total number of operations performed in different layers. As the complete model is not required at the time of inference, the complexity is calculated using the layers required for inference, i.e., the encoder layers, the multi-size split layers, the edge feature extractor layers, upscaling and merge, and the clustering layer. The inference time of our network is about 60–80 ms, which makes our method fast enough to be applied for live imaging applications. Validating this, we have used our method to detect edges for live acquisition of depth maps using an Intel RealSense 3D camera. Figure 10 shows some screenshots captured for two different scenes during this process. More detailed results can be found in the supplementary video.

## Conclusion

In this paper, we have proposed an unsupervised deep learning-based method for 3D edge detection, and parameter selection for the depth maps of organised point clouds. We have formulated the edge detection problem as a clustering problem, where edges are clustered as edge and non-edge, based on automatically selected threshold values. The optimal cluster distributions are obtained using *k-means* clustering technique. The automatic thresholding process overcomes a key challenge associated with other state-of-the-art methods, i.e., manual tuning of algorithm parameters and the requirement for labelled training data. We have compared the performance of our approach with four other methods, over five different datasets, in terms of pose registration accuracy and F-measure. The results of these experiments demonstrate that our approach exhibits comparative performance to state-of-the-art methods, without the need for any manual tuning of thresholds or the necessity of accurately labelled data. In the future, we plan to use our method for real-world robotic visual servoing, grasping and manipulation tasks. In particular, we plan to extend our method to perform model matching for 3D pose estimation and real-time tracking.

### Supplementary Information

Supplementary Information.

## Data Availability

Datasets used for analysis in this study will be available to share upon request to the corresponding author.

## Code availability

Custom code developed for this study and for result reproduction will be available to share upon request to the corresponding author.
